# AJP001, a novel helper T‐cell epitope, induces a humoral immune response with activation of innate immunity when included in a peptide vaccine

**DOI:** 10.1096/fba.2019-00056

**Published:** 2019-11-22

**Authors:** Akiko Tenma, Hironori Nakagami, Hideki Tomioka, Makoto Sakaguchi, Ryoko Ide, Hiroshi Koriyama, Hiroki Hayashi, Munehisa Shimamura, Hiromi Rakugi, Ryuichi Morishita

**Affiliations:** ^1^ Department of Health Development and Medicine Osaka University Graduate School of Medicine Osaka Japan; ^2^ FunPep Co Osaka Japan; ^3^ Department of Neurology Osaka University Graduate School of Medicine Osaka Japan; ^4^ Department of Geriatric and General Medicine Osaka University Graduate School of Medicine Osaka Japan; ^5^ Department of Clinical Gene Therapy Osaka University Graduate School of Medicine Osaka Japan

**Keywords:** peptide, T‐cell epitope, vaccine

## Abstract

Vaccine design requires well‐tailored formulations including a T‐cell epitope and adjuvants. We identified a novel cationic peptide, AJP001, which possesses a strong affinity for murine MHC class II alleles (H2‐IE^d^ and H2‐IA^d^) and low affinity for H2‐IA^b^. We designed an AJP001 and epitope peptide‐conjugated vaccine, AJP001‐angiotensin (Ang) II, which was intracutaneously administered to mice three times at 2‐week intervals. Indeed, the AJP001‐Ang II vaccine induced antibody production against Ang II in BALB/cA mice but not in C57BL/6 mice. To estimate the T‐cell‐dependent immunogenicity of the AJP001 conjugate vaccine in human cells, naïve human peripheral blood mononuclear cells (PBMCs) were exposed to AJP001‐Ang II, and T‐cell proliferation was evaluated by analyzing cell division using flow cytometric measurement of carboxyfluorescein succinimidyl ester (CFSE) dye dilution. To activate the immune response, the innate immune system must be activated by adjuvant treatment. Interestingly, treatment with AJP001 induced IL‐1β and IL‐18 secretion via NLRP3 inflammasome activation and induced TNF‐α and IL‐6 production through an NF‐κB‐dependent pathway in human and mouse macrophages. These results suggest that AJP001 behaves as a T‐cell epitope in mice and humans and is a useful tool for the formulation of peptide vaccines without the addition of adjuvants.

**Abbreviations**
Ang II: Angiotensin II
BMDC: Bone marrow‐derived dendritic cell
CD: cluster of differentiation
CFSE: carboxyfluorescein succinimidyl ester
DT: diphtheria toxoid
ELISpot: enzyme‐linked immunospot
HBsAg: hepatitis B virus surface antigen
HLA‐DR: Human Leukocyte Antigen ‐ DR isotype
ICAM‐1: intercellular adhesion molecule‐1
IEDB: The Immune Epitope Database
KLH: keyhole limpet hemocyanin
MFI: Mean Fluorescence Intensity
MHC: major histocompatibility complex
MVF: measles virus fusion protein
MyD88: Myeloid differentiation primary response 88
NF‐κB: Nuclear factor kappa B
NLRP3: NACHT, LRR, and PYD domains‐containing protein 3
PBMC: peripheral blood mononuclear cell
PMA: phorbol‐12‐myristate‐13‐acetate
PT: pertussis toxin
RFI: relative fluorescence intensity
siRNA: small interfering RNA
TLR: Toll‐like receptor
TRIF: TIR‐domain‐containing adapter‐inducing interferon‐β
TT: tetanus toxoid
VLP: virus‐like particle

## 1 INTRODUCTION

Current vaccine design requires careful procedures, selective antigens and formulations including T‐cell epitopes and adjuvants. In the design of B‐cell‐type peptide vaccines, B‐cell epitopes are usually conjugated to large carrier proteins, such as keyhole limpet hemocyanin (KLH), virus‐like particle particles (VLP), tetanus toxoid (TT), or diphtheria toxoid (DT).^1^ Because large carrier proteins are highly immunogenic, they enable the induction of antibody production against coupled B‐cell epitopes. However, this approach is fraught with difficulties in controlling the uniformity of the coupling process and provoking undesirable immune responses such as allergy and anaphylaxis. In recent years, chimeric peptide vaccines composed of B‐cell epitopes and T‐cell epitopes have been developed and studied in clinical trials to evaluate the effectiveness of these vaccines.^2^, ^3^, ^4^ In this strategy, the T‐cell epitope is MHC class II restricted; hence, it should be promiscuous or universal, allowing broad population coverage, and is required to include a helper T‐cell epitope to elicit specific T cells and humoral responses. Furthermore, to efficiently induce antibody production via T‐cell activation by vaccines, cotreatment with adjuvants contributes to the activation of an innate immune response to break down immune tolerance through the activation of Toll‐Like Receptors (TLRs), Retinoic acid‐Inducible Gene‐I (RIG‐I), or inflammasomes.^5^, ^6^ Alum is a well‐known adjuvant that drives a Th2‐biased immune response and induces the release of endogenous danger signals, also called “alarmins”, via localized cellular damage,^7^ and these alarmins directly stimulate inflammasomes via NLRP3.^8^

We previously developed an antimicrobial peptide, termed angiogenic peptide 30 (AG30), with a length of 30 amino acids that possesses both angiogenic and antibacterial functions 9, 10, 11 similar to the functions of LL‐37 and PR39.^12^, ^13^ We further designed and synthesized a series of AG30 analogs and identified a candidate adjuvant peptide (AJP001), which strongly induced the activation of inflammasomes and the NF‐κB pathway. An analysis using tools in The Immune Epitope Database (IEDB) showed that the AJP001 peptide potentially possesses a helper T‐cell epitope. Because it is required to include a helper T‐cell epitope to elicit specific T cell and humoral responses and to induce the activation of innate immunity in the formulation of chimeric peptide vaccines, the potency of AJP001 has been evaluated by analyzing humoral immune responses in mice and in human cells.

## 2 MATERIALS AND METHODS

### 2.1 Materials

The Ang II and AJP001 conjugated vaccine (AJP001‐Ang II), Ang II and BSA conjugate (BSA‐Ang II), DPP4 epitope peptide and AJP001 conjugated vaccine and LL‐37 were synthesized by the Peptide Institute, Inc. AJP001, AJP406, and magainin‐2 were synthesized by ILS Inc. Ang II, LPS, and PMA were obtained from Sigma‐Aldrich (St. Louis, USA). Alum (Alhydrogel^®^ 2%, aluminum hydroxide gel) was obtained from InvivoGen. CpG oligodeoxynucleotides (2006) were obtained from Novus Biologicals. Monoclonal mouse anti‐human CD54 and ICAM‐1/FITC (Clone 6.5B5) antibodies were obtained from Dako Denmark A/S. FITC‐conjugated mouse anti‐human CD86 (Clone FUN‐1), FITC‐conjugated mouse IgG1κ isotype control, APC‐conjugated mouse anti‐human CD3, and PE‐Cy7‐conjugated mouse anti‐human CD4 antibodies, and 7‐AAD were obtained from BD Pharmingen. The CD4^+^ T‐cell Isolation Kit human was obtained from Miltenyi Biotec. The CellTrace CFSE Cell Proliferation Kit was obtained from Life Technologies Corporation. Anti‐human NLRP3 and anti‐human β‐actin antibodies were obtained from Cell Signaling Technology, Inc. NLRP3‐specific siRNAs (FlexiTube siRNA Hs_CIAS1_6 and Hs_CIAS1_9), a control siRNA (AllStars Negative Control siRNA) and HiPerFect Transfection Reagent were obtained from QIAGEN. QNZ and BAY11‐7082 were obtained from Enzo Life Sciences, Inc. Ca‐074‐Me was obtained from the Peptide Institute, Inc Z‐YVAD‐FMK was obtained from BioVision. Mouse IFN‐γ ELISpot Development Module, Mouse IL‐4 ELISpot Development Module and ELISpot Blue Color Module were obtained from R&D Systems, Inc.

### 2.2 Cells and cell culture

The THP‐1 cell line was obtained from the JCRB Cell Bank and maintained in RPMI 1640 medium (Invitrogen) supplemented with 10% (vol/vol) fetal bovine serum (Invitrogen) and 0.05 mmol/L 2‐mercaptoethanol. The RAW264.7 cell line was obtained from the American Type Culture Collection and cultured in DMEM (Invitrogen) supplemented with 10% (vol/vol) fetal bovine serum. All cultures were maintained at 37°C in a humidified 5% CO_2_ incubator. Human PBMCs were commercially obtained from Cellular Technology Limited (OH, USA), which was approved to use only for the experiment, and cultured in Optimizer medium supplemented with Optimizer T‐cell expansion supplement and 2 mM GlutaMax (Gibco/Life technologies).

### 2.3 LPS priming and PMA differentiation

For cell priming, cells were incubated with 1 μg/mL of LPS in RPMI 1640 medium for 3 hours (for THP‐1 cells) or with 50 ng/mL of LPS in DMEM for 3 hours (for RAW264.7 cells). After incubation with LPS, cells were washed with fresh medium and used in the cytokine production assay. To induce cell differentiation, cells were seeded in RPMI 1640 medium supplemented with 50 ng/mL of PMA for 48 hours. After incubation, the PMA‐containing medium was removed, and adherent cells were washed once with fresh medium followed by AJP001 treatment.

### 2.4 Animal studies and immunization

All experiments were approved by the Ethical Committee for Animal Experiments of the Osaka University Graduate School of Medicine. TLR4 KO, MyD88 KO, TLR9 KO, and TRIF KO C57BL/6 mice were purchased from OrientalBioService, Inc. Six‐week‐old female BALB/cA and male C57BL/6 mice were purchased from CLEA Japan, Inc. Six‐week old male DIS/Eis (Dahl‐Iwai S) rats were purchased from Japan SLC, Inc and housed in a temperature‐ and light cycle‐controlled facility with free access to food and water. AJP001, AJP001‐Ang II, or AJP001‐DPP4‐1 were dissolved in saline and were injected intracutaneously on days 0, 14, and 28. Serum was collected from the tail vein, and antibody titers against immunized peptides were determined by ELISA. Immunization Study of AJP001‐DPP4‐2, 3, 4 were conducted at KAC Co., Ltd and complied with the guidelines on animal care of the local Ethics Committee on the Use of Animals. AJP001‐DPP4 vaccines were immunized intracutaneously at 2 weeks interval and boosted at 9 weeks after first immunization. The peptide sequences were shown in Table S1.

### 2.5 h‐CLAT assay

The h‐CLAT assay was performed as described previously.^14^, ^15^ THP‐1 cells were seeded at a density of 2 × 10^5^ cells/mL and precultured for 48 hours in RPMI 1640 medium supplemented with 10% (vol/vol) fetal bovine serum and 0.05 mmol/L 2‐mercaptoethanol. After incubation, the cells were collected by centrifugation and resuspended in RPMI 1640 medium at a density of 2 × 10^6^ cells/mL; 500 µL was seeded in a 24‐well plate before the treatment. Then, 500 µL of diluted peptides or alum and medium control were added to the cells and incubated for 24 hours.

After treatment for 24 hours, the fluorescence intensities of the THP‐1 cell surface markers were analyzed by flow cytometry (FACS Canto II). Cell staining was performed using FITC‐conjugated anti‐human CD54 (clone; 6.5B5), FITC‐conjugated anti‐human CD86 (clone; FUN‐1), and FITC‐labeled mouse IgG1κ isotype control. Cells were incubated with the above mAbs for 30 minutes at 4°C. After washing and resuspending in PBS supplemented with 0.1% BSA, analysis was performed by flow cytometry. Propidium iodide solution (Sigma) was used at a concentration of 0.625 μg/mL. After the dead cells were gated out, a total of 10 000 living cells were analyzed. When the cell viability was less than 50%, the relative fluorescence intensity (RFI) was not calculated. RFI was used as an indicator of CD86 and CD54 expression and was calculated by the following formula: RFI (%) = (MFI of peptide‐treated cells − MFI of peptide‐treated isotype control cells)/(MFI of vehicle control cells − MFI of vehicle isotype control cells) × 100 MFI = (geometric) mean fluorescence intensity.

### 2.6 NLRP3 expression knockdown in THP‐1 cells

First, 100 nmol/L of NLRP3 siRNA (Hs_CIAS1_6 and Hs_CIAS1_9) or control siRNA were transfected into THP‐1 cells using HiPerFect Transfection Reagent. After overnight culture, siRNA‐transfected THP‐1 cells were primed with LPS followed by AJP001 stimulation. The NLRP3 knockdown efficiency was analyzed by Western blotting.

### 2.7 Western blot analysis

The siRNA‐transfected cell lysates were prepared with RIPA lysis buffer (Santa Cruz Biotechnology) supplemented with 2 mmol/L PMSF, 1 mmol/L Na_3_VO_4_, and proteinase inhibitor cocktail. The protein concentration was determined for each sample, and then equal amounts of protein were subjected to SDS/PAGE. After electrophoresis, the membrane was incubated with anti‐human NLRP3 antibody or anti‐human β‐actin antibody. After incubation with HRP‐conjugated antibodies specific for rabbit IgG (GE Healthcare), chemiluminescence signal was detected with a Luminescent Image Analyzer (LAS‐1000, FUJIFILM) and analyzed with MultiGauge version 3.2 software.

### 2.8 Bone marrow‐derived dendritic cell preparation

Bone marrow‐derived dendritic cells (BMDCs) were generated as previously described.^16^, ^17^ Briefly, bone marrow was flushed from femurs and tibias of wild‐type, TLR4 KO, MyD88 KO, TLR9 KO, or TRIF KO mice and seeded at a final cell density of 2 × 10^5^ cells/mL into 100 mm culture dishes in RPMI 1640 medium supplemented with 20 ng/mL of rmGM‐CSF (R&D Systems, Inc), 10% (vol/vol) fetal bovine serum, and 0.05 mmol/L 2‐mercaptoethanol. On day 3 of the culture, 10 mL of fresh culture medium with 20 ng/mL rmGM‐CSF was added to each dish. On day 6 of the culture, nonadherent cells were collected and used for the cytokine production assay. BMDCs were seeded in 24‐well plates, and diluted AJP001 or LPS was added to each well and cultured for 24 hours. After incubation, the culture supernatant was collected, and the IL‐1β concentration in the culture supernatant was determined by ELISA (R&D Systems, Inc).

### 2.9 Cytokine production assay

THP‐1 cells and RAW264.7 cells were seeded in 24‐well plates, and diluted AJP001, CpG, and alum were added to each well and cultured for 24 hours. After incubation, the culture supernatant was collected, and IL‐1β, IL‐18, TNF‐α, and IL‐6 concentrations in the culture supernatant were determined by ELISA (R&D Systems, Inc). For the inhibition assay, QNZ, BAY11‐7082 (NF‐κB inhibitors), Ca‐074‐Me (cathepsin B inhibitor), and Z‐YVAD‐FMK (caspase‐1 inhibitor) were pretreated for 30 minutes before treatment with AJP001.

### 2.10 Measurement of antibody titers by ELISA

ELISA plates (MaxiSorp Nunc; Thermo Fisher Scientific) were coated with 10 μg/mL of BSA‐Ang II or AJP001 in carbonate buffer overnight at 4°C. After blocking with PBS containing 5% skim milk (blocking buffer), the sera were diluted 10‐ to 31,250‐fold in blocking buffer. After incubation overnight at 4°C, the plates were washed with PBS‐T and then incubated with horseradish peroxidase (HRP)‐conjugated anti‐mouse IgG antibody (GE Healthcare) for at least 3 hours at room temperature. After washing with PBS‐T, color development with the peroxidase chromogenic substrate 3,3′‐5,5′‐tetramethyl benzidine (Sigma‐Aldrich) was performed, and the reaction was stopped by the addition of 0.5 N sulfuric acid. The absorbance at 450 nm was detected using a microplate reader (iMark Microplate Absorbance Reader, Bio‐Rad Laboratories, Inc). The half‐maximal antibody titer was determined according to the highest value in the dilution range of each sample.

### 2.11 ELISpot assay

The 96‐well ELISpot plates (Millipore) were coated with anti‐mouse IFN‐γ capture antibody or anti‐mouse IL‐4 capture antibody overnight at 4°C. After incubation, the plates were washed with wash buffer (PBS containing 0.05% Tween 20) and then blocked with 1% BSA and 5% sucrose in PBS. Splenocytes were isolated from immunized mice and added to the plates (5 × 10^5^ cells per well) and stimulated with 10 μg/mL of Ang II or AJP001 peptide at 37°C for 48 hours. PMA and ionomycin were added as positive controls at a concentration of 100 ng/mL each. The plates were washed with wash buffer followed by incubation with biotinylated anti‐mouse IFN‐γ or IL‐4 antibody overnight at 4°C. After washing, streptavidin‐alkaline phosphatase was added to each well and incubated for 2 hours at room temperature. After washing with wash buffer and rinsing with deionized water, plates were incubated with 5‐bromo‐4‐chloro‐3′‐indolyl phosphate p‐toluidine salt (BCIP) and Nitro Blue Tetrazolium solution (NBT) for 30 minutes at room temperature. Finally, plates were rinsed with deionized water and air‐dried at room temperature. Spots were counted using a stereo microscope (Stemi 305, Carl Zeiss Microscopy Co., Ltd).

### 2.12 T‐cell proliferation induced by AJP001 in human PBMCs in vitro

For the estimation of T‐cell‐dependent immunogenicity of the AJP001 conjugate vaccine in humans, a T‐cell proliferation assay in human PBMCs was performed as described with a small modification.^18^ Cryopreserved PBMCs were thawed and incubated with 0.2 μmol/L of CFSE in PBS at 37°C for 5 minutes. Staining was terminated by adding FBS containing Medium, the cells were washed once and suspended in Optimizer medium supplemented with Optimizer T‐cell expansion supplement, 2 mmol/L GlutaMax (Gibco). Stained cells were cultured in 96‐well round bottom plates with medium alone or with AJP001‐Ang II or KLH (2 × 10^5^ cells/100 μL/well). After 7‐9 days of culture, cells for each antigen concentration were pooled, washed with PBS and stained with Zombie Violet dye at room temperature for 15 min to exclude dead cells and followed by staining with anti‐CD3 APC‐conjugated and anti‐CD4 PE‐Cy7‐conjugated antibodies. Cells were analyzed by a FACS Canto II flow cytometer and FACSDiva software (BD Biosciences). The percentage of proliferating T cells was measured by determining the percentage of CFSE^low^ cells in the CD3‐ and CD4‐positive cell populations.

### 2.13 Statistical analyses

All data are expressed as the mean ± SD. Differences between two groups were assessed using an unpaired two‐tailed Student's *t*‐test. Data sets involving more than two groups were assessed by Dunnett's test using GraphPad Prism version 6.07 (GraphPad Software). A difference was considered statistically significant at *P* < .05.

## 3 RESULTS

### 3.1 AJP001 induces CD86 and CD54 expression in THP‐1 cells

**Figure 1:**
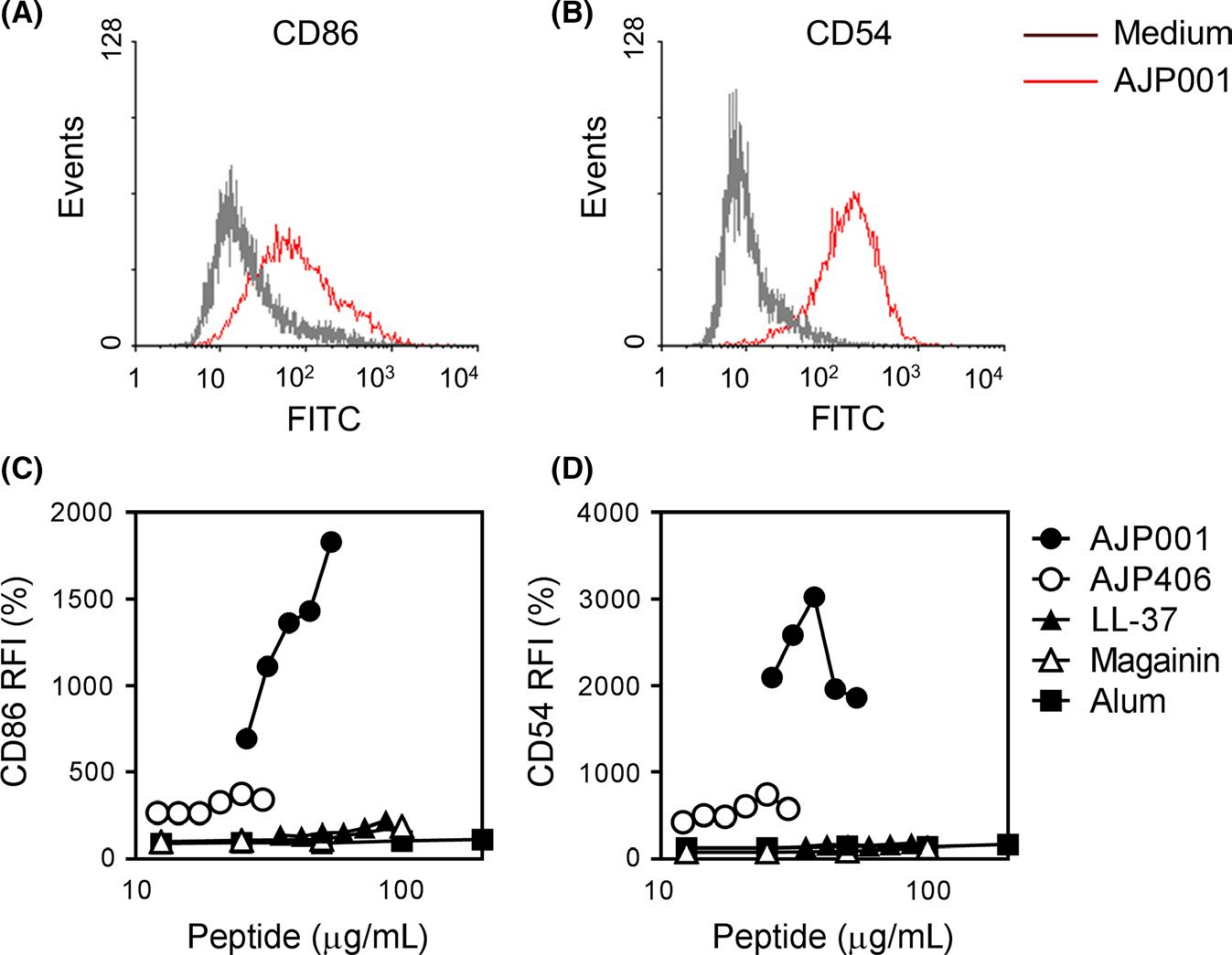
AJP001 induces CD86 and CD54 expression in THP‐1 cells. A, B. Representative histogram analyses show CD86 or CD54 expression (x‐axis) versus the cell count (y‐axis). THP‐1 cells were treated with AJP001 (20 μg/mL) overnight, and the expression of CD86 and CD54 was measured by flow cytometry. C, D. The expression of human CD86 and CD54 was measured by flow cytometry. THP‐1 cells were treated with AJP001, AJP406, LL‐37, magainin or Alum overnight. RFI was calculated as follows: RFI (%) = (MFI of peptide‐treated cells − MFI of peptide‐treated isotype control cells)/(MFI of vehicle control cells − MFI of vehicle isotype control cells) × 100.

We previously developed a novel functional peptide, AG30 that possesses both angiogenic and antibacterial functions.^9^, ^10^, ^11^ To move this peptide toward clinical application, we evaluated several candidate peptides by modifying AG30 to enhance its function and stability. Through the process of peptide screening, the skin sensitization potency of these peptides was evaluated by the human Cell Line Activation Test (h‐CLAT), which is a toxicity screening method that measures CD86 and CD54 expression in THP‐1 cells.^14^, ^15^ As shown in Table 1, two candidate peptides (AJP001 and AJP406) and two known antimicrobial peptides (LL‐37 and magainin‐2) were examined. Treatment with AJP001 markedly increased the expression of CD86 and CD54 in a dose‐dependent manner (Figure 1A,B). Although AJP406 treatment also induced CD86 and CD54 expression, AJP406 potency was much weaker than AJP001 potency. LL‐37 and magainin‐2, which are well‐known antimicrobial peptides, did not possess any activity (Figure 1C,D). These results indicated that AJP001 is a potent stimulator of the expression of the costimulatory molecule CD86 and adhesion molecule CD54/ intercellular adhesion molecule (ICAM)‐1.

**Table 1:** Amino acid sequence of AG30‐modified peptides

Peptide	Amino acid sequence
AG30/5C	MLKLIFLHRLKRMRKRLKRKLRLWHRKRYK
AJP406	GRLKRLGERLKRKIQKLIRL‐amide
AJP001	Ac‐ELKLIFLHRLKRLRKRLKRK‐amide
LL‐37	LLGDFFRKSKEKIGKEFKRIVQRIKDFLRNLVPRTES
Magainin‐2	GIGKFLHSAKKFGKAFVGEIMNS

### 3.2 Prediction of the MHC class II binding affinity of AJP001

We further evaluated the potency of AJP001 for the development of peptide vaccine, which requires the inclusion of a helper T‐cell epitope to elicit specific T cell and humoral responses. The strength of the binding affinity between a peptide and an MHC molecule in antigen‐presenting cells (APCs) may predict the potency of helper T‐cell activation, which leads to antibody production in B cells. To confirm whether the AJP001 peptide has a helper T‐cell epitope, the binding affinity of AJP001 for murine MHC class II alleles (H2‐IE^d^, H2‐IA^d^ and H2‐IA^b^) was analyzed using the IEDB (http://www.iedb.org/home_v3.php) MHC II binding prediction tool. The 15‐mer helper T‐cell epitopes of AJP001 were predicted, and the results are shown as percentile ranks (Table 2). Lower percentile ranks correspond to higher binding affinities for MHC class II alleles. AJP001 showed a strong affinity for H2‐IE^d^ with percentile ranks from 4.6 to 7.5 and a high affinity for H2‐IA^d^ with percentile ranks from 15.4 to 30.7. Since the haplotype of BALB/cA mice includes H2‐IE^d^ and H2‐IA^d^, AJP001 could potentially induce helper T cells in BALB/cA mice. In contrast, AJP001 showed a low affinity for H2‐IA^b^ with percentile ranks ranging from 81.0 to 87.0. Since the haplotype of C57BL/6 mice includes H2‐IA^b^, AJP001 might not activate helper T cells in C57BL/6 mice.

**Table 2:** Prediction of major histocompatibility complex (MHC) Class II binding affinity of AJP001 using IEDB MHC II binding prediction tool

Allele	Start	End	Sequence	Percentile rank
H2‐IE^d^	6	20	FLHRLKRLRKRLKRK	4.6
	4	18	LIFLHRLKRLRKRLK	5.3
	3	17	KLIFLHRLKRLRKRL	5.7
	5	19	IFLHRLKRLRKRLKR	5.7
	2	16	LKLIFLHRLKRLRKR	6.1
	1	15	ELKLIFLHRLKRLRK	7.5
H2‐IA^d^	5	19	IFLHRLKRLRKRLKR	15.4
	6	20	FLHRLKRLRKRLKRK	15.8
	4	18	LIFLHRLKRLRKRLK	21.6
	2	16	LKLIFLHRLKRLRKR	21.9
	3	17	KLIFLHRLKRLRKRL	25.9
	1	15	ELKLIFLHRLKRLRK	30.7
H2‐IA^b^	1	15	ELKLIFLHRLKRLRK	81.0
	2	16	LKLIFLHRLKRLRKR	81.3
	3	17	KLIFLHRLKRLRKRL	82.7
	4	18	LIFLHRLKRLRKRLK	84.5
	5	19	IFLHRLKRLRKRLKR	85.8
	6	20	FLHRLKRLRKRLKRK	87.0

### 3.3 Vaccination with AJP001 induces antibody production and T‐cell activation in mice

**Figure 2:**
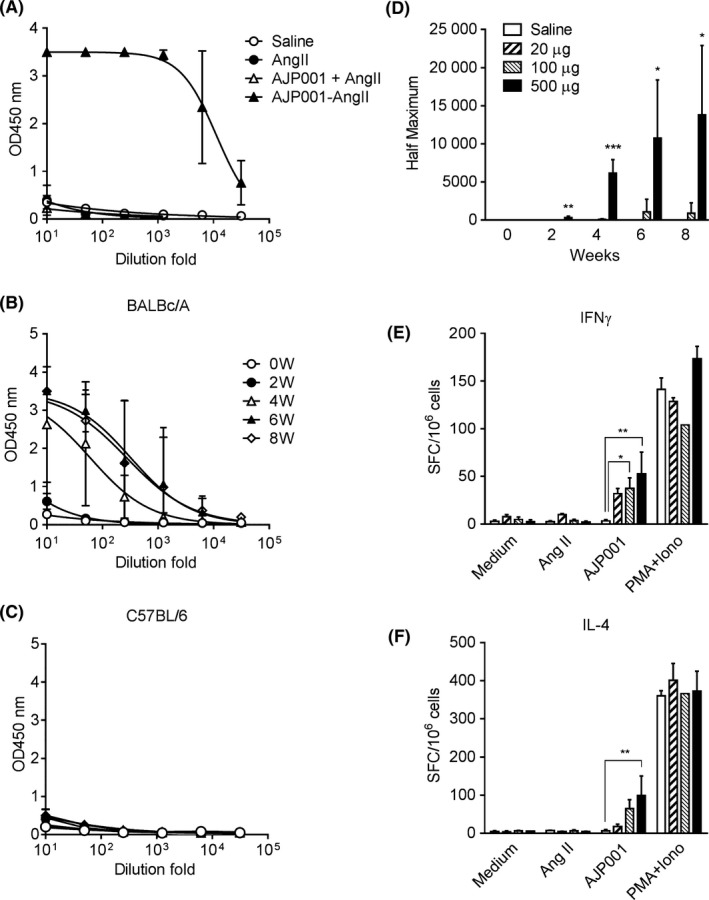
Vaccination with AJP001 induces antibody production and T‐cell activation in mice. A. Ang II, a mixture of Ang II and AJP001 or the AJP001‐Ang II conjugate vaccine was administered intracutaneously to 7‐week‐old female BALB/cA three times at 2‐week intervals. The anti‐Ang II IgG antibody titer in serum samples collected at 0, 2, 4, 6, and 8 weeks was detected by ELISA. The data represent the mean OD at 450 nm and the SD at each serum dilution fold (n = 3). B, C. The AJP001‐Ang II conjugate vaccine (AJP001‐Ang II 100 μg) was administered intracutaneously to 7‐week‐old female BALB/cA (B) or 7‐week‐old male C57BL/6 (C) mice three times at 2‐week intervals without any adjuvant cotreatment. The anti‐Ang II IgG antibody titer in serum sample collected at 0, 2, 4, 6, and 8 weeks was detected by ELISA. The data represent the mean OD at 450 nm and the SD at each serum dilution fold (n = 3). D. The AJP001‐Ang II conjugate vaccine was administered intracutaneously to 7‐week‐old female BALB/cA mice at a dose of 20, 100, or 500 μg per mouse three times at 2‐week intervals without any adjuvant cotreatment. The anti‐Ang II IgG antibody titer in serum samples collected at 0, 2, 4, 6, and 8 weeks was measured by ELISA. The titers are expressed as the dilution fold of the serum giving half‐maximal absorbance at 450 nm. All data are expressed as the mean ± SD (n = 3). *P < .05, **P < .01 and ***P < .001 vs the saline group. E, F. Antigen‐specific activation of T cells in AJP001‐Ang II‐immunized mice was evaluated by an ELISpot assay. Splenocytes were isolated from AJP001‐Ang II‐immunized mice and stimulated with Angiotensin II or AJP001 at a concentration of 10 μg/mL. PMA and ionomycin (100 ng/m each) were added to positive control wells, and medium was added as a negative control. The number of IFN‐γ‐ (E) or IL‐4‐producing (F) cells was detected by counting spots using a stereomicroscope. The number of spots was quantified in the duplicate or triplicate wells of each mouse. The data represent the mean ± SD (n = 3). *P < .05 and **P < .01 vs the saline group

To evaluate the immunogenicity of AJP001 in vivo, we designed an AJP001 and epitope peptide‐conjugated vaccine, which involved the conjugation of both peptides spaced by ε‐Acp (Table S1). AJP001 was conjugated to angiotensin (Ang) II (AJP001‐Ang II) because it has been reported that the Ang II peptide does not include a helper T‐cell epitope and that the Ang II peptide vaccine successfully induces anti‐Ang II antibody production.^19^, ^20^ The conjugated peptide vaccine was intracutaneously administered to BALB/cA mice at a dose of 500 μg/mouse. To assess the antibody‐inducing capacity of AJP001‐Ang II, Ang II was administered alone (140 μg/mouse) or in combination with AJP001 (360 μg/mouse) in formulations containing the same amount of each molecule as that found in 500 μg of AJP001‐Ang II. The titers of antibodies against Ang II in the serum were measured by enzyme‐linked immunosorbent assay (ELISA). According to the results, only AJP001‐Ang II induced anti‐Ang II antibody production (Figure 2A). This result suggests that AJP001 functions as a T‐cell epitope when conjugated with an antigenic epitope. Furthermore, we compared the immunogenicity of AJP001 in different strains of mice. AJP001‐Ang II was intracutaneously administered to BALB/cA and C57BL/6 mice three times at 2‐week intervals at a dose of 100 μg/mouse. The AJP001‐Ang II vaccine induced antibody production against Ang II in the BALB/cA mice (Figure 2B) but not in the C57BL/6 mice (Figure 2C). It is assumed that the difference in antibody production between the two strains involved the binding affinity of AJP001 for their MHC class II alleles. According to the MHC class II binding predictions (Table 2), AJP001 revealed a higher affinity for the alleles of the BALB/cA mice (H2‐IE^d^ and H2‐IA^d^) than for the allele of the C57BL/6 mice (H2‐IA^b^); hence, antibody responses corresponded to the binding affinity predictions for AJP001. Furthermore, we evaluated the dose responsiveness of the antibody production induced by the AJP001‐Ang II vaccine and the T‐cell activation in immunized mice by enzyme‐linked immunospot (ELISpot) assay. The AJP001‐Ang II vaccine was intracutaneously administered to BALB/cA mice three times at 2‐week intervals at a dose of 20, 100, or 500 μg/mouse without adjuvant cotreatment. The AJP001‐Ang II vaccine induced antibody production against Ang II in a dose‐dependent manner in the BALB/cA mice (Figure 2D). After three immunizations, splenocytes were isolated from the immunized mice, and the antigen‐specific production of interferon (IFN)‐γ and interleukin (IL)‐4 was evaluated using an ELISpot assay (Figure 2E,F). Stimulation with AJP001 induced the production of IFN‐γ and IL‐4 in a dose‐dependent manner in vivo. However, Ang II did not elicit any response in the ELISpot assay. These results indicate that AJP001 is an adequate T‐cell epitope to induce T‐cell activation in immunized mice.

### 3.4 AJP001‐DPP4 epitope conjugated vaccines induce antibody production in mice and AJP‐Ang II induces antibody in rats

To confirm antibody inducing ability of AJP001 for another antigen, B‐cell epitope peptides of DPP4 and AJP001‐conjugated peptide were synthesized.^21^ The conjugated peptide vaccine (AJP001‐DPP4‐1) was intracutaneously administered to BALB/cA mice three times at 2‐week intervals at a dose of 100 μg/mouse and anti‐DPP4 IgG antibody was detected at 6 and 8 weeks after first administration (Figure S1A). Additionally, three different sequences of epitope peptides were designed (AJP001‐DPP4‐2, 3, 4) and immunized with the same administration schedule as AJP001‐DPP4‐1 and boosted at 9 weeks (The peptide sequences were shown in Table S1). Anti‐DPP4 antibody production was detected from 2 weeks after first immunization and a boost effect was observed after the fourth administration in AJP001‐DPP4‐2 immunized mouse serum (Figure S1B). However, AJP001‐DPP4‐3 and 4 induced low anti‐DPP4 antibody response, hence the antibody titer induced AJP001‐conjugated vaccine was affected by B‐cell epitope sequence.

In order to confirm antibody inducing ability of AJP001 in other species, we utilized Dahl rats with AJP001‐conjugate vaccine. Antibody production elicited by AJP001‐conjugated vaccine was confirmed in AJP001‐Ang II immunized Dahl rats (Figure S1C).

### 3.5 T‐cell proliferation induced by AJP001 in human PBMCs in vitro

**Figure 3:**
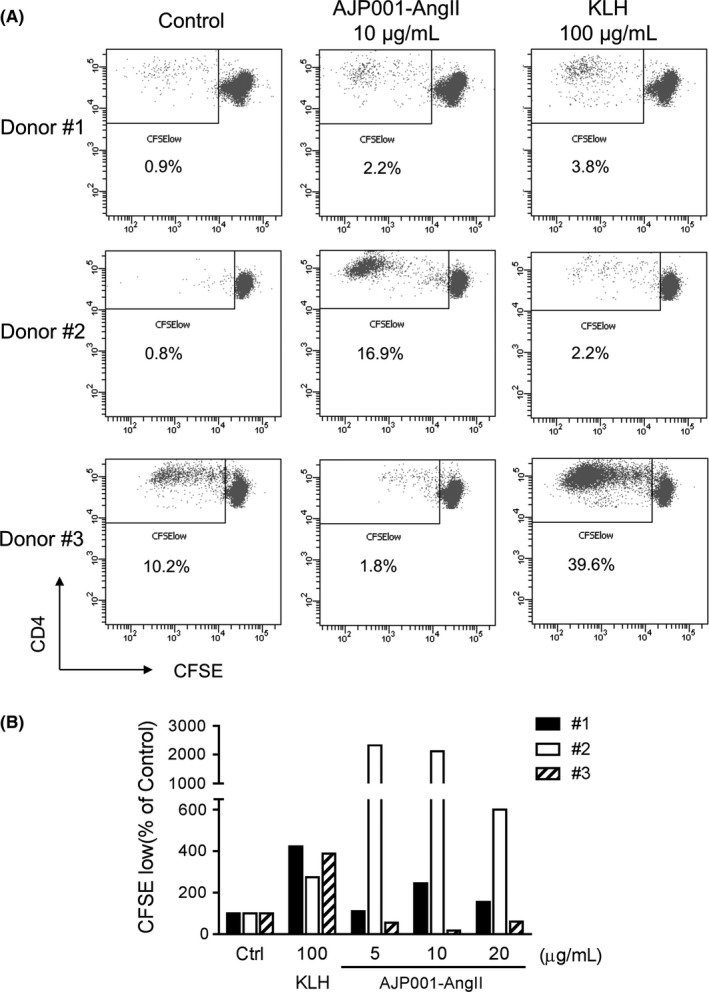
T‐cell proliferation induced by AJP001‐Ang II in human PBMCs in vitro. Naïve human PBMCs were labeled with CFSE and cultured for 7‐9 days upon stimulation with AJP001‐Ang II (5, 10, and 20 μg/mL) or KLH (100 μg/mL), and CFSE fluorescence was assessed by flow cytometry. The percentage of proliferating T cells was measured by determining the percentage of CFSE^low^ cells in CD3‐ and CD4‐positive cell populations. A. Representative FACS plots showing CFSE dilution in CD3+ and CD4+ T‐cells stimulated with AJP001‐Ang II (10 μg/mL) or KLH (100 μg/mL) and not stimulated (Control). B. Proliferation of T cell in AJP001‐Ang II or KLH treated cells were calculated as percentage of control (medium alone) cells

To evaluate whether AJP001 is capable of inducing T‐cell proliferation in humans, naïve human peripheral blood mononuclear cells (PBMCs) were stimulated with AJP001‐Ang II, and helper T‐cell proliferation was assessed by analyzing cell division using flow cytometric measurement of carboxyfluorescein succinimidyl ester (CFSE) dye dilution. AJP001‐Ang II induced strong T‐cell proliferation in donor #2 and moderate in donor #1. No proliferation was observed in donor #3 and KLH showed T‐cell proliferation in all donors (Figure 3). AJP001 binding affinity to HLA‐DRB allele of each donor was predicted by IEDB server (Figure S2). AJP001 demonstrated extremely strong affinity to HLA‐DRB1*11:01 of donor #2 and low affinity to HLA‐DRB1*01:01 and DRB1*13:02 of donor #3. T‐cell proliferation activity of AJP001‐Ang II was apparently correlated with predicted HLA‐DRB binding affinity of AJP001. These results indicate that AJP001 is a T‐cell epitope that is able to induce T‐cell responses in humans.

### 3.6 Cytokine production induced by AJP001 in LPS‐primed THP‐1 cells

**Figure 4:**
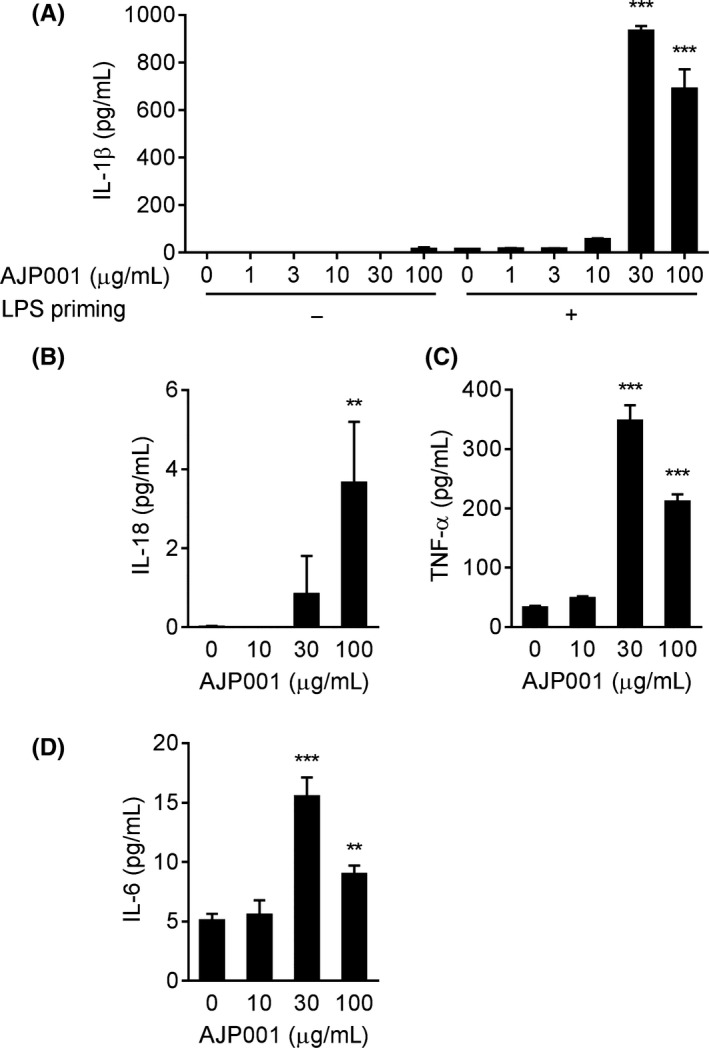
AJP001 induces cytokine production in LPS‐primed THP‐1 cells. A. IL‐1β production was measured by ELISA. THP‐1 cells were primed with 1 μg/mL LPS‐containing medium for 3 hours or incubated with cell culture medium and subsequently incubated with AJP001 (1, 3, 10, 30, or 100 μg/mL) or medium overnight. B‐D. Human IL‐18, TNF‐α, and IL‐6 production was measured by ELISA. THP‐1 cells were primed with 1 μg/mL LPS‐containing medium for 3 hours and subsequently incubated with AJP001 (10, 30, or 100 μg/mL) or medium overnight. Data represent the mean + SD (n = 3). **P < .01 and ***P < .001 vs 0 μg/mL AJP001

Peptide vaccines designed to induce humoral responses usually require the activation of innate immunity in addition to helper T cells. As shown in Figure 1, AJP001 treatment significantly induced the expression of CD86 and CD54/ICAM‐1, which is an important ability involved in an adjuvant potentiating the immunogenicity of antigens through the activation of innate immunity.^10^ To examine the mechanism underlying the activation of innate immunity, we addressed the inflammasome pathway and NF‐κB pathway. First, the involvement of AJP001 in inflammasome activation was examined in THP‐1 cells. IL‐1β release by THP‐1 cells requires two distinct signals; first, pro‐IL‐1β synthesis is induced by priming with lipopolysaccharide (LPS) or phorbol‐12‐myristate‐13‐acetate (PMA), and then the activation of inflammasomes leads to the maturation and secretion of IL‐1β, which is a process conducted by activated caspase‐1. THP‐1 cells were primed with LPS (1 μg/mL) for 3 hours, followed by stimulation with AJP001 overnight. AJP001 induced IL‐1β secretion in a dose‐dependent manner only in the LPS‐primed THP‐1 cells (Figure 4A). Similarly, IL‐18 was also produced by the LPS‐primed THP‐1 cells exposed to AJP001, which indicated that AJP001 was associated with caspase‐1 activation in inflammasomes (Figure 4B). In addition, the production of the inflammatory cytokines TNF‐α and IL‐6 was also significantly increased by AJP001 treatment (Figure 4C,D). Interestingly, increases in TNF‐α and IL‐6 production were observed in THP‐1 cells that were not primed with LPS (data not shown), but the maximum levels of TNF‐α and IL‐6 production were different from those of IL‐1β and IL‐18 production. Similar results were obtained in PMA‐stimulated THP‐1 cells (Figure S2). These results suggest that AJP001 treatment increases both inflammasome‐dependent cytokine secretion (IL‐1β and IL‐18) and inflammasome‐independent cytokine production (TNF‐α and IL‐6).

### 3.7 AJP001 activates NLRP3 inflammasomes

**Figure 5:**
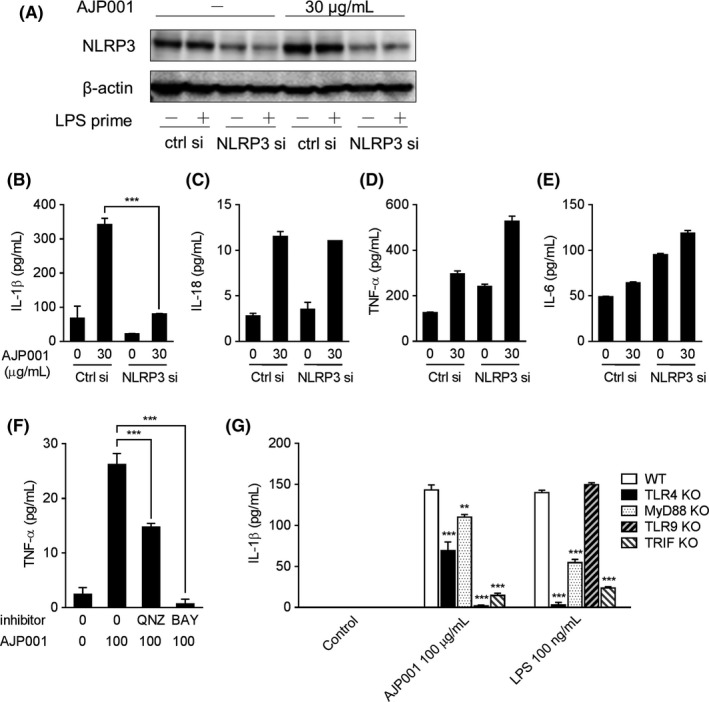
AJP001 activates inflammasomes and NF‐κB in THP‐1 cells. A. NLRP3 depletion in THP‐1 cells was detected by western blotting. THP‐1 cells were transfected with an NLRP3‐specific siRNA or a control siRNA for 24 hours. The siRNA‐transfected cells were primed with 1 μg/mL LPS‐containing medium for 3 hours and subsequently incubated with AJP001 (30 μg/mL) overnight. B‐E. Human IL‐1β, IL‐18, TNF‐α, and IL‐6 production were measured by ELISA with or without NLRP3 deletion. The NLRP3‐specific siRNA‐ or control siRNA‐transfected cells were primed with LPS, followed by incubation with AJP001 (30 μg/mL) overnight. Data represent the mean + SD (n = 3). ***P < .001. F. Human TNF‐α production was measured by ELISA. THP‐1 cells were pretreated with the NF‐κB inhibitors QNZ (10 μmol/L) and BAY11‐7082 (10 μmol/L), followed by incubation with AJP001 (100 μg/mL) or medium overnight. Data represent the mean + SD (n = 3). ***P < .001. G. AJP001‐induced IL‐1β production is TLR9 dependent. BMDCs from wild‐type, TLR4‐KO, MyD88‐KO, TLR9‐KO or TRIF‐KO mice were cultured with AJP001 (100 μg/mL), LPS (100 ng/mL) or medium for 24 hours. IL‐1β production by the BMDCs was measured by ELISA. Data represent the mean + SD (n = 3). **P < .01 and ***P < .001 vs the control

To investigate whether inflammasome activation is mediated by NLRP3, we tested inflammatory cytokine production in NLRP3‐knockdown THP‐1 cells. An NLRP3‐specific or a control siRNA was transfected into THP‐1 cells, which were then cultured for 24 hours. The NLRP3‐knockdown THP‐1 cells were primed with LPS for 3 hours and then stimulated with 30 μg/mL AJP001 for 24 hours. Inflammatory cytokines in the culture supernatant were measured by ELISA. We confirmed that NLRP3 expression was decreased in THP‐1 cells by the NLRP3‐specific siRNA (Figure 5A). The induction of IL‐1β production by AJP001 was significantly decreased by the knockdown of NLRP3 expression (Figure 5B). Unexpectedly, IL‐18 production was not affected by NLRP3 knockdown (Figure 5C). These results indicate that the induction of IL‐1β secretion by AJP001 might be mediated by NLRP3. The increase in TNF‐α and IL‐6 production induced by AJP001 was also unaffected by NLRP3 silencing (Figure 5D,E). Therefore, a further investigation was performed using the cathepsin B inhibitor Ca‐074‐Me and the caspase‐1 inhibitor Z‐YVAD‐FMK. IL‐1β and IL‐18 secretion in response to AJP001 was significantly inhibited by the addition of Ca‐074‐Me (10 μmol/L) or Z‐YVAD‐FMK (10 μmol/L) (Figure S3A,B), whereas TNF‐α production was not affected by treatment with these inhibitors (Figure S3C). These results suggest that the production of IL‐1β and IL‐18 by AJP001 is dependent on the NLRP3 activation associated with lysosomal cathepsin release and caspase‐1 activation by NLRP3, while the TNF‐α production induced by AJP001 is mediated by another pathway.

### 3.8 AJP001 activates the NF‐κB pathway and CD86 and CD54/ICAM‐1 expression

To confirm whether the TNF‐α and IL‐6 production induced by AJP001 might be involved in NF‐κB activation, we next used NF‐κB inhibitors. The TNF‐α production induced in THP‐1 cells by AJP001 treatment was significantly inhibited by QNZ and BAY11‐7082 (Figure 5F). These results indicate that AJP001 activates the NF‐κB pathway and induces TNF‐α and IL‐6 production. We further analyzed the contribution of TLR pathways to AJP001‐induced NF‐κB activation; primary cell cultures from TLR4‐, myeloid differentiation primary response (MyD)88‐, TLR9‐, or TIR domain‐containing adaptor protein inducing IFN‐β (TRIF)‐deficient mice were prepared to evaluate the effects of AJP001 and LPS on IL‐1β expression (Figure 5G). The effect of AJP001 was diminished in the cultures from the TLR9‐ and TRIF‐KO mice and, to a lesser extent, in those from the TLR4‐ and MyD88‐KO mice, whereas the effect of LPS was diminished in the cultures from the TLR4‐ and TRIF‐KO mice and, to a lesser extent, in those from the MyD88‐KO mice but not in those from the TLR9‐KO mice. These results suggest that AJP001 activates TLR4 and TLR9, leading to NF‐κB activation.

Next, we compared the effect of AJP001 on the expression of the costimulatory molecule CD86 and the adhesion molecule CD54/ICAM‐1 with that of alum. Although AJP001 is a potent stimulator of CD86 and CD54/ICAM‐1 expression, alum did not increase the expression of these molecules at all (Figure 1C,D). Thus, treatment with AJP001 resulted in CD86/CD54 expression, which might be beyond the effects of alum.

### 3.9 Cytokine production induced by AJP001 in RAW264.7 cells

To confirm the effect of AJP001 on mice, the involvement of AJP001 in inflammasome activation was examined in murine macrophage cells (RAW264.7 cells). RAW264.7 cells were primed with LPS (50 ng/mL) for 3 hours, followed by stimulation with AJP001 overnight. AJP001 significantly induced mouse IL‐1β secretion in a dose‐dependent manner only in the LPS‐primed RAW264.7 cells (Figure S4A). Similarly, mouse IL‐18, TNF‐α, and IL‐6 were also produced by the LPS‐primed RAW264.7 cells exposed to AJP001 (Figure S4B‐D). These results suggest that AJP001 treatment increases both inflammasome‐dependent cytokine secretion (IL‐1β and IL‐18) and inflammasome‐independent cytokine production (TNF‐α and IL‐6) in mice.

## 4 DISCUSSION

In this study, AJP001 showed a strong affinity for the haplotype of BALB/cA mice, and AJP001 stimulation increased inflammatory cytokine levels via NLRP3 activation and NF‐κB activation.^8^ Indeed, the antibody‐producing efficiency of the AJP001 conjugate vaccine showed that AJP001 contained a T‐cell epitope that was adequate for inducing T‐cell activation in immunized mice.

We and others have recently focused on therapeutic vaccines to induce a humoral immune response, not a cytotoxic response, to accelerate antibody production. For example, Ang II vaccine for hypertension consists of KLH‐conjugated Ang II and an adjuvant to induce anti‐Ang II antibody production.^19^, ^20^ These B‐cell‐type peptide vaccines are required to be conjugated to large carrier proteins such as KLH.^1^ For clinical application, chimeric peptide vaccines composed of a B‐cell epitope and T‐cell epitope have been developed instead of vaccines including large carrier proteins.^2^ In this study, AJP001 showed a strong affinity for the haplotype of BALB/cA mice, and the antibody‐producing efficiency of the AJP001 conjugate vaccine correlated with its affinity. In addition, binding affinity for human HLA‐DR was predicted, and AJP001 revealed a strong binding affinity for HLA‐DR alleles that are frequently found in the Japanese population (data not shown). Indeed, in a T‐cell proliferation assay with human PBMCs, AJP001 induced peptide‐specific T‐cell activation and demonstrated potency as an applicable T‐cell epitope peptide for human use. Human MHC class II (HLA class II) molecules exhibit extensive variation; therefore, the T‐cell epitope of a peptide vaccine for human application should be promiscuous to cover a broad range of HLA class II haplotypes. In previous reports, UBITh® was shown to have helper T‐cell epitopes that comprise a collection of promiscuous helper T‐cell epitopes directly derived from the highly antigenic proteins of pathogens, such as measles virus fusion protein (MVF), hepatitis B virus surface antigen (HBsAg), TT, and pertussis toxin (PT).^22^ Of importance, AJP001 possesses innate immune‐activating activity and helper T‐cell epitopes. This advantage allows us to create a simple formulation for our peptide vaccine without adding adjuvants.

**Figure 6:**
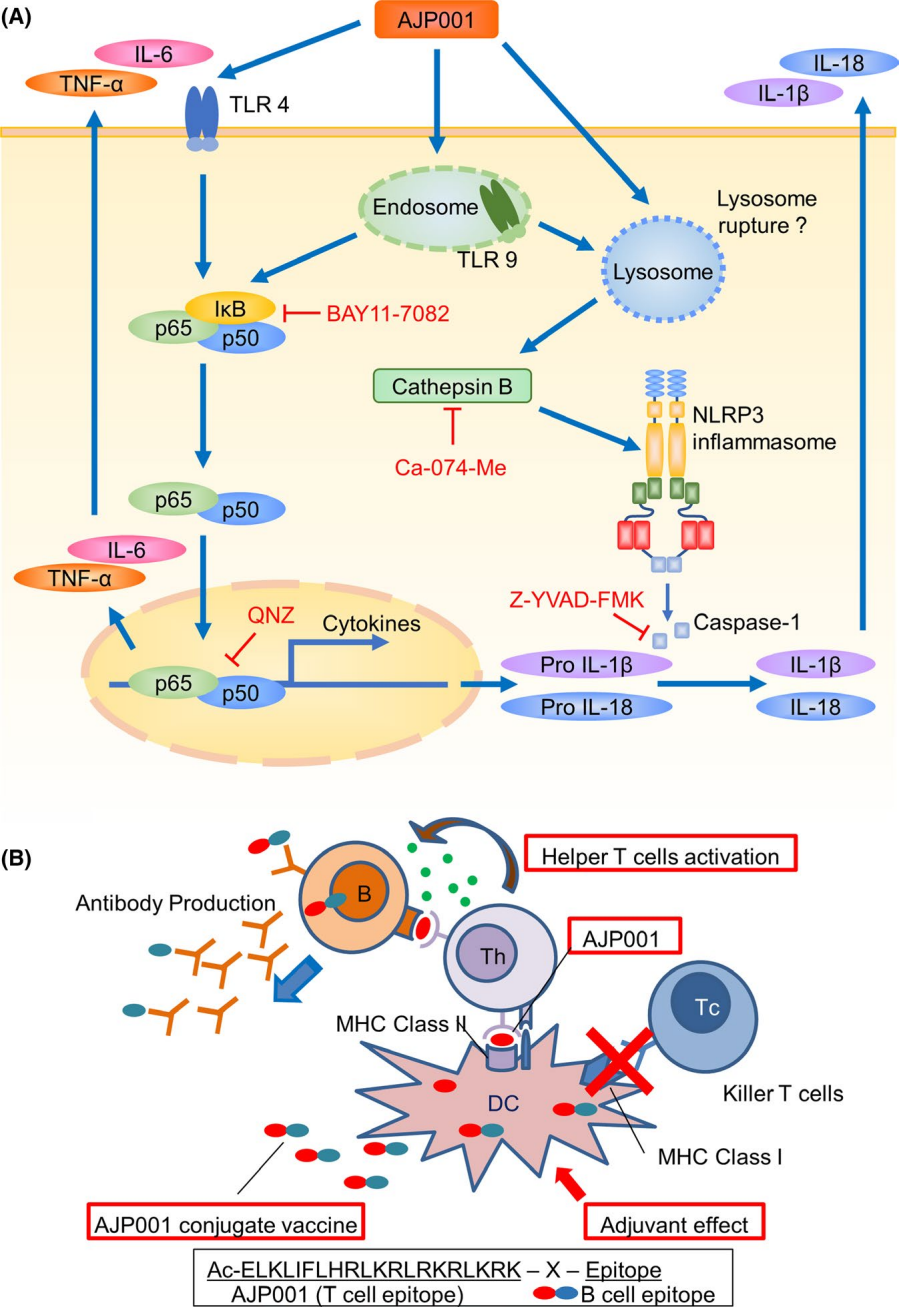
Scheme of predicted AJP001 signaling and the antibody production induced by the AJP001 conjugate peptide vaccine. A. AJP001 activates TLR4 and TLR9 to activate the NF‐κB signaling pathway, which results in an increase in the expression of several inflammatory cytokines (ie, TNF‐α and IL‐6). AJP001 may induce cathepsin B release from lysosomes, leading to the activation of NLRP3 inflammasomes; however, the mechanism of cathepsin B release into the cytoplasm is unclear. Caspase‐1, a component of the NLRP3 inflammasome, is activated by cathepsin B and processes pro‐IL‐1β and pro‐IL‐18 into their mature forms, which are released from the cell. IL‐1β and IL‐18 production is dependent on cathepsin B and the NLRP3 inflammasome pathway because this production is inhibited by a cathepsin B inhibitor (Ca‐074‐Me) and caspase‐1 inhibitor (Z‐YVAD‐FMK). B. The AJP001 conjugate peptide vaccine stimulates helper T (Th) cells and induces epitope‐specific antibody production in B cells. The AJP001‐B‐cell epitope conjugate peptide vaccine is taken up by antigen‐presenting cells (APCs), such as dendritic cells and B cells. AJP001 or a peptide containing the AJP001 peptide sequence is presented on the cell surface in association with an MHC class II molecule. AJP001 elicits adjuvant effects and induces the production of cytokines and expression of costimulatory molecules such as CD86. Naïve CD4+T cells (Th) that have a TCR that binds to the AJP001‐MHC class II complex are activated with the costimulatory molecules induced by AJP001. Activated Th cells recognize B cells presenting AJP001 bound to MHC class II; subsequently, the B cells are activated and induced to produce antibody

AJP001 strongly increased the expression of NF‐κB‐dependent inflammatory cytokines (ie, IL‐6 and TNF‐α), which are similar to those induced by TLR ligands. Indeed, the effect of AJP001 was diminished in primary cell cultures using cells from TLR4‐ or TLR9‐deficient mice. As shown in Figure 6, AJP001 may also induce cathepsin B release from lysosomes, leading to the activation of NLRP3 inflammasomes. Caspase‐1, a component of the NLRP3 inflammasome, is activated by cathepsin B and processes pro‐IL‐1β and pro‐IL‐18 into their mature forms, which are released from the cell. IL‐1β and IL‐18 production is dependent on cathepsin B and the NLRP3 inflammasome pathway. These results suggest that AJP001 potentially interacts with multiple signaling pathways to activate the innate immune response. Although no antimicrobial peptides have been known to be direct stimulants of TLRs, LL‐37, a human antimicrobial peptide, in combination with DNA strongly induces IFN‐α and IFN‐β via TLR9.^23^, ^24^ Recently, LL‐37 and poly (I:C) were also found to synergistically induce the expression of IFN‐β via TLR3, a sensor of double‐stranded RNA.^25^ Although a close relationship between TLRs and this antimicrobial peptide could be speculated, we hypothesized that AJP001 activates TLR9 directly or in combination with self‐DNA released from the host cell.

Overall, we identified a novel peptide, AJP001, that possessed strong activity for inducing CD86 and CD54 expression and inflammasome and NF‐κB pathway activation, leading to adjuvant action and potent induction of T‐cell activation in humans. Thus, AJP001 is a novel adjuvant and T‐cell epitope‐containing peptide that is useful in a conjugate vaccine for the induction of a humoral immune response.

## CONFLICT OF INTEREST

The Department of Clinical Gene Therapy is financially supported by Novartis, AnGes, Shionogi, Boeringher, Fancl, Saisei Mirai Clinics, Rohto, and FunPep. The Department of Health Development and Medicine is financially supported by AnGes, Dycel, FunPep, and Mitsubishi‐Tanabe. R. Morishita is a stockholder of FunPep and Anges. A. Tenma, H. Tomioka, M. Sakaguchi, and R. Ide are employees of FunPep. R. Morishita, H. Tomioka, and A. Tenma are FunPep stockholders. The funder provided support in the form of salaries for authors but did not have any additional role in the study design, data collection and analysis, decision to publish, or preparation of the manuscript.

## AUTHOR CONTRIBUTIONS

A. Tenma, M. Sakaguchi, R. Ide, H. Koriyama, H. Hayashi, and M. Shimamura conducted the experiments and acquired data. A. Tenma and H. Nakagami designed the experiment and analyzed the data in discussion with H. Tomioka, H. Rakugi and R. Morishita. A. Tenma and H. Nakagami wrote the manuscript. All authors read and commented on this manuscript.
