# Supplementary material for: AJP001, a novel helper T‐cell epitope, induces a humoral immune response with activation of innate immunity when included in a peptide vaccine
Source: FASEB Bioadv. 2019 Nov 22;1(12):760–72. doi: 10.1096/fba.2019-00056 (PMC6996369; doi:10.1096/fba.2019-00056)
Supplement: Supplementary file 1 [file FBA2-1-760-s001.pdf]

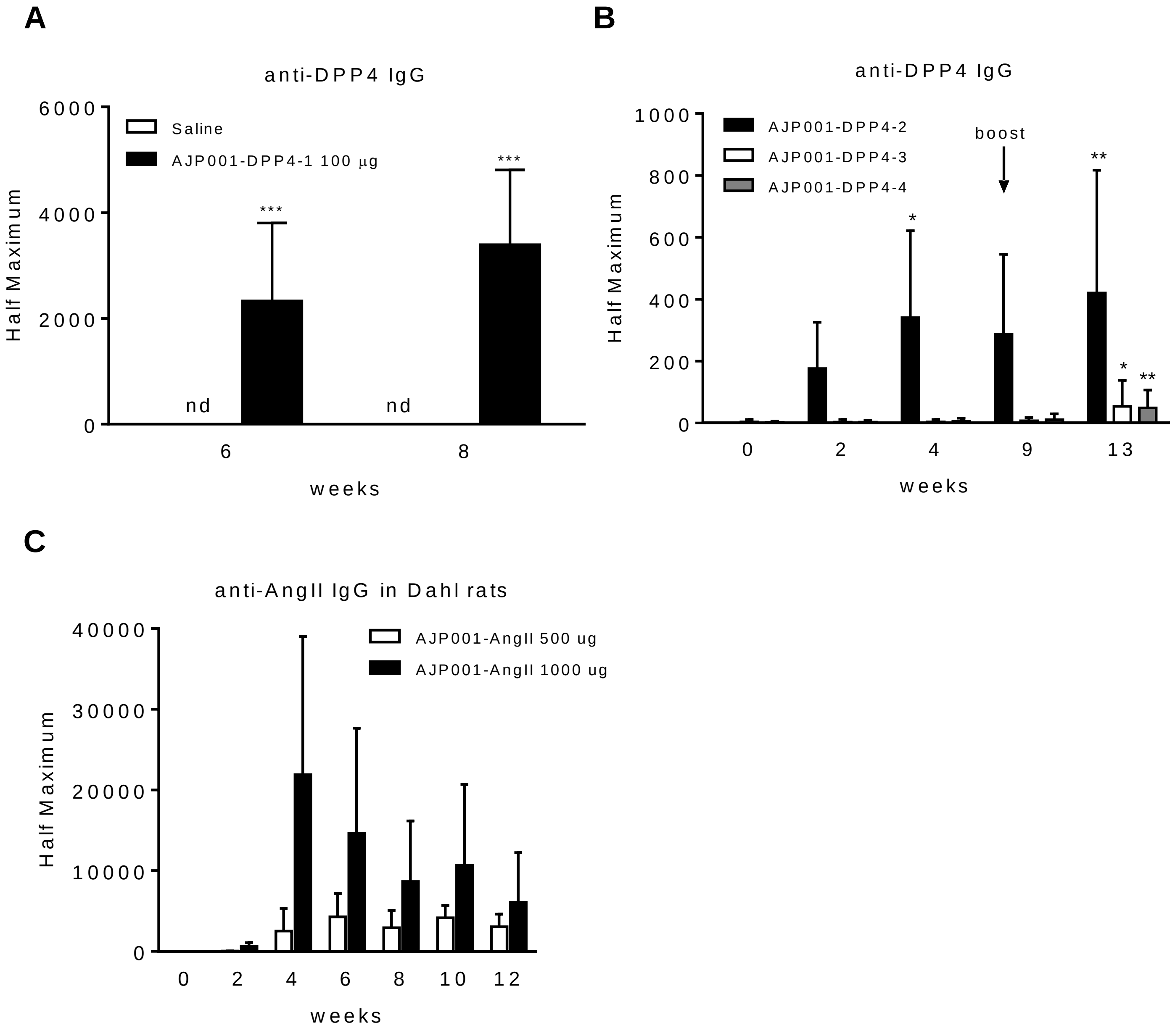

Supplemental Figure 1.

(A) AJP001-DPP4-1 epitope conjugated vaccine was administered intracutaneously to BALB/cA mice at a dose of 100 μg per mouse three times at two-week intervals (n=6). (B) AJP001-DPP4-2,3,4 epitope conjugated vaccines were administered intracutaneously to BALB/cA mice at a dose of 250 μg per mouse at 0, 2 and 4 weeks and boosted at 9 weeks (n=8). (C) AJP001-Ang II was administered intracutaneously to Dahl rats at a dose of 500 or 1000 μg per rat at 0, 2 and 4 weeks (n=3). The anti-DPP4 IgG antibody titer in serum samples was measured by ELISA. The titers are expressed as the dilution fold of the serum giving half-maximal absorbance at 450 nm. All data are expressed as the mean ± SD. (A) ***$P < 0.001$ vs the saline group analyzed by t-test. (B) *$P < 0.05$ and **$P < 0.01$ vs 0 week analyzed by Dunnett's test.

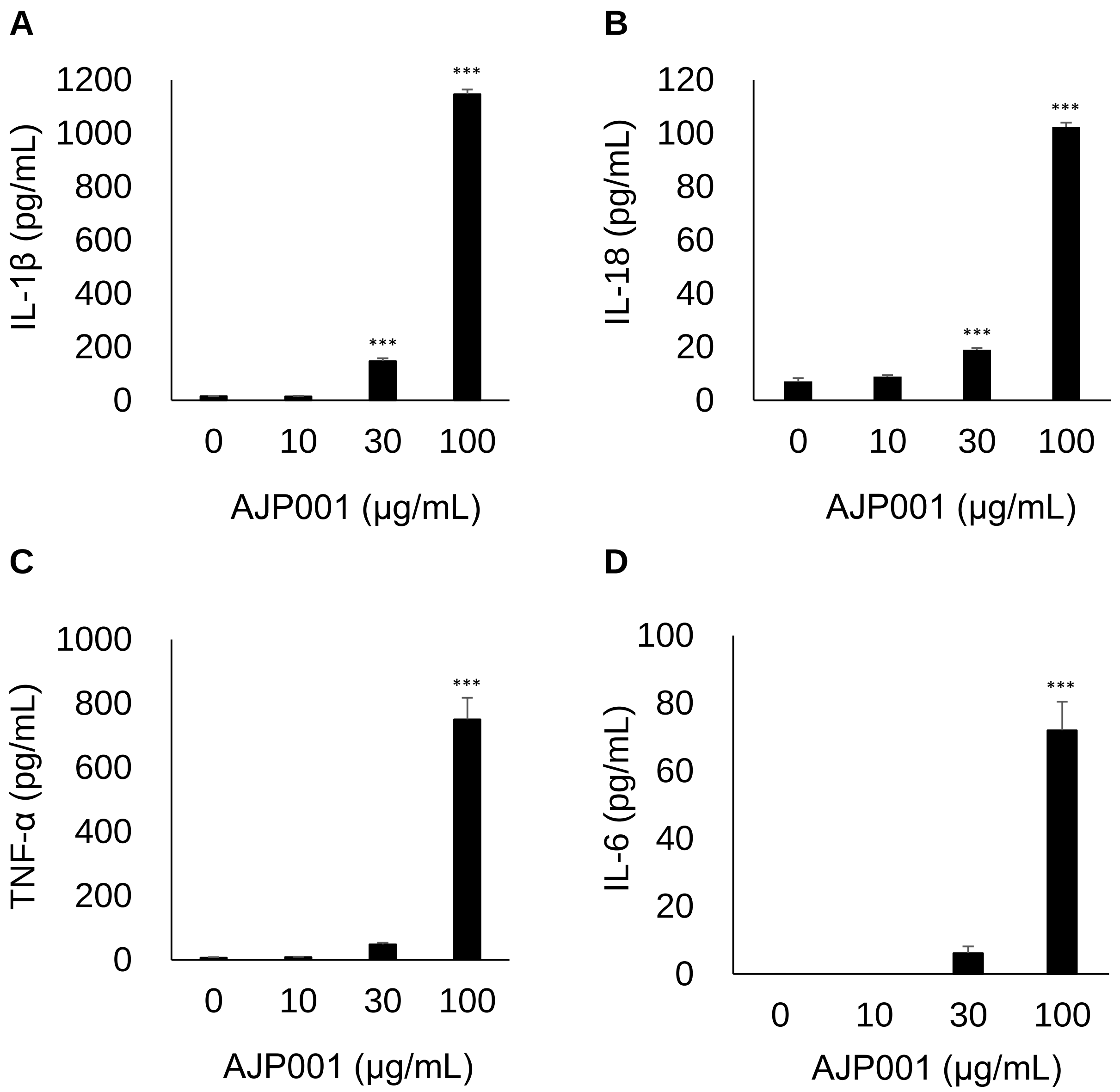

Supplemental Figure 2.

Cytokine production induced by AJP001 in PMA differentiated THP-1 cells.

AJP001 induced cytokine production in differentiated THP-1 cells.

Human IL-1β (A), IL-18 (B), TNF-α (C) and IL-6 (D) production were measured by ELISA. THP-1 cells were differentiated into macrophage with 50 ng/mL of PMA for 2 days and subsequently incubated with AJP001 (10, 30 and 100 µg/mL) or medium overnight. Data represent mean + SD (n=3). *** : $p < 0.001$

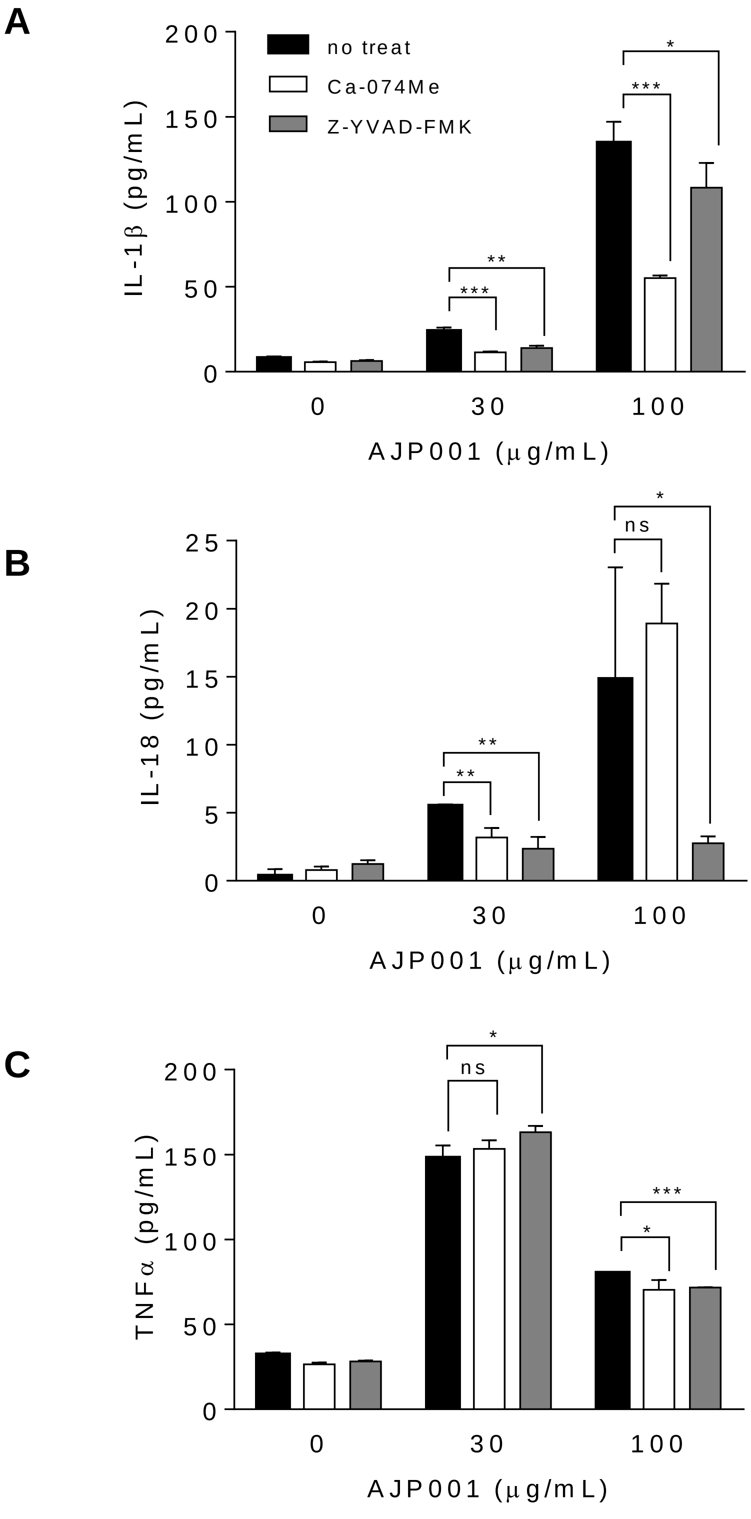

Supplemental Figure 3.

Effect of Cathepsin B inhibitor (Ca-074Me) and caspase-1 inhibitor (Z-YVAD-FMK) on AJP001-induced inflammasome activation in THP-1 cells.

Human IL-1β (A), IL-18 (B) and TNF-α (C) production were measured by ELISA. THP-1 cells were primed with 1 μg/mL of LPS-containing medium for 3 h. Primed cells were incubated with Ca-074Me (10μM) or Z-YVAD-FMK (10μM) for 30min followed by AJP001 treatment (10, 30 and 100 μg/mL) or medium overnight. Data represent mean + SD (n=3). *** : $p < 0.001$, ** : $p < 0.01$, * : $p < 0.05$.

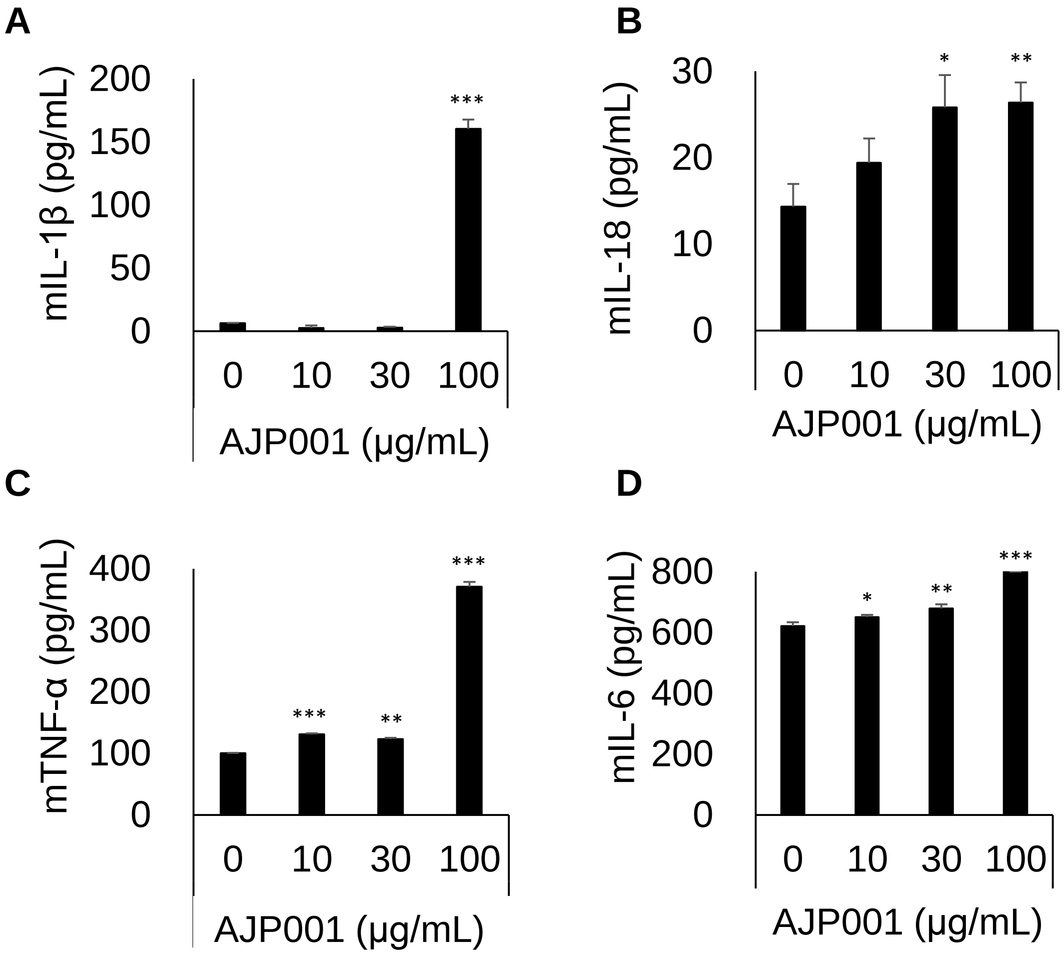

Supplemental Figure 4.
Cytokine Production induced by AJP001 in RAW264.7 cells
Mouse IL-1β (A), IL-18 (B), TNF-α (C) and IL-6 (D) production were measured by ELISA. RAW264.7 cells were primed with 50 ng/mL of LPS-containing medium for 3 h and subsequently incubated with AJP001 (10, 30 and 100 μg/mL) or medium overnight. Data represent mean + SD (n=3). * : $p < 0.05$, ** : $p < 0.01$, *** : $p < 0.001$ vs AJP001 0 μg/mL

Supplemental Table 1. Amino acid sequence of AJP001-conjugated peptide vaccines

Peptide vaccine	Amino acid sequence
AJP001-AngII	Ac-ELKLIFLHRLKRLRKRLKRK-X*-DRVYIHPF
AJP001-DPP4-1	Ac-ELKLIFLHRLKRLRKRLKRK-X-ENSTFESFG
AJP001-DPP4-2	Ac-ELKLIFLHRLKRLRKRLKRK-X-PPHFDKSKKY - amide
AJP001-DPP4-3	Ac-ELKLIFLHRLKRLRKRLKRK-X-NSSVFLENSTFDEFG - amide
AJP001-DPP4-4	Ac-ELKLIFLHRLKRLRKRLKRK-X-NSSIFLENSTFESFG - amide

*: X=ε-Acp

Supplemental Table 2. Prediction of HLA-DRB binding affinity of AJP001 using IEDB MHC II binding prediction tool.

Donor	DRB1	
	Allele	Percentile rank
#1	03:01	12.0
	15:01	4.30
#2	11:01	0.09
	15:01	4.30
#3	01:01	19.0
	13:02	13.0
